# Too Much Surgery

**Published:** 1888-02

**Authors:** 


Too Much Surgery.—Dr. C. D. Palmer, of Cincinnati, read a paper at the last meeting of the American Gynecological Society, on "The Therapeutic Value of some Medicines in the Treatment of Hemorrhagic Conditions of the Uterus." After referring to the fact that the work of the Society had been largely surgical, only five papers on the therapeutical uses of medicine and three on obstetric subjects having been read before it, and also to the fact that a similar state of affairs existed in all the medical and surgical societies in this country, the author of the paper said that uterine hemorrhage could often be treated advantageously by means of drugs. Is comment necessary?

